# Purification and Characterization of Two Novel Laccases from *Peniophora lycii*

**DOI:** 10.3390/jof6040340

**Published:** 2020-12-06

**Authors:** Olga A. Glazunova, Konstantin V. Moiseenko, Olga S. Savinova, Tatyana V. Fedorova

**Affiliations:** A.N. Bach Institute of Biochemistry, Research Center of Biotechnology of the Russian Academy of Sciences, 119071 Moscow, Russia; mr.moiseenko@gmail.com (K.V.M.); savinova_os@rambler.ru (O.S.S.)

**Keywords:** laccase, *Peniophora lycii*, substrate specificity, dye decolorization

## Abstract

Although, currently, more than 100 laccases have been purified from basidiomycete fungi, the majority of these laccases were obtained from fungi of the Polyporales order, and only scarce data are available about the laccases from other fungi. In this article, laccase production by the white-rot basidiomycete fungus *Peniophora lycii*, belonging to the Russulales order, was investigated. It was shown that, under copper induction, this fungus secreted three different laccase isozymes. Two laccase isozymes—Lac5 and LacA—were purified and their corresponding nucleotide sequences were determined. Both purified laccases were relatively thermostable with periods of half-life at 70 °C of 10 and 8 min for Lac5 and LacA, respectively. The laccases demonstrated the highest activity toward ABTS (97 U·mg^−1^ for Lac5 and 121 U·mg^−1^ for LacA at pH 4.5); Lac5 demonstrated the lowest activity toward 2,6-DMP (2.5 U·mg^−1^ at pH 4.5), while LacA demonstrated this towards gallic acid (1.4 U·mg^−1^ at pH 4.5). Both Lac5 and LacA were able to efficiently decolorize such dyes as RBBR and Bromcresol Green. Additionally, phylogenetic relationships among laccases of *Peniophora* spp. were reconstructed, and groups of orthologous genes were determined. Based on these groups, all currently available data about laccases of *Peniophora* spp. were systematized.

## 1. Introduction

Fungi that cause white rot of wood (i.e., white-rotting fungi or white-rot fungi) are a unique group of decomposers that can degrade all components of plant cell walls, including such a difficultly degradable biopolymers as lignin. In nature, these fungi play a fundamental role in carbon balance, soil formation, and forest regeneration [1,2]. Almost all white-rot fungi belong to the Basidiomycota division; ascomycetes of the genus *Xylaria* are an exception. In the process of growth and development, white-rot fungi produce a vast array of extracellular enzymes, one of which—laccase—has been attracting the attention of researchers for more than 100 years [3].

Laccase (benzenediol:oxygen oxidoreductase, EC 1.10.3.2) is a multi-copper oxidase that can perform one-electron oxidation of different substrates with a concomitant reduction of molecular oxygen to water [4]. As an oxidizing substrate, laccases can utilize substituted phenols, aromatic amines, and a number of other complex aromatic compounds containing hydroxyl or amino groups [5]. Due to their broad substrate specificity, laccases can be potentially used in a wide variety of technical applications, including pulp and paper and textile industries, production of biosensors, wastewater treatment, and soil bioremediation [6,7,8].

Although laccase is an enzyme with more than 100 years’ history of exploration, its exact biological function is still debatable. Originally, laccase was considered as a lignin-degrading enzyme, but later this view was reassessed [9]. Over the past decades, several biological functions were proposed for laccases including morphogenesis, fungal plant–pathogen/host interaction, stress defense, pigment formation, and detoxification of phenolic compounds [10,11]. Additionally, it was speculated that in different fungi, laccases can perform different functions; however, current genomic data had demonstrated that the situation is more complicated. Based on the extensive sequencing, performed under the 1000 Fungal Genomes Project [12], it was demonstrated that laccase genes in basidiomycete genomes always form multigene families comprised of many non-allelic (paralogous, serially duplicated) genes. Consequently, each fungus can produce many laccase isozymes (i.e., products of different non-allelic genes) that can perform different functions. From the above, it is apparent that only systematic comparisons of laccases from different fungi, as well as comparisons of laccase isozymes from the same fungus, can potentially shed light on the range of biological functions of laccases. In addition, the more naturally occurring variations in laccase are discovered, the better rational design of this enzyme can be performed for biotechnology.

The majority of currently characterized laccases were obtained from fungi of the Polyporales order. Recently, evolutionary relationships among these laccases were reconstructed, and their orthology-based classification was proposed [13]. At least for the Core Polyporoid clade of Polyporales, this classification was proven to be useful for bringing order to the very scattered data regarding different laccase isozymes from different fungi. Additionally, information about the orthology relationships between genes allows invoking so-called “orthology conjecture”—i.e., potential extrapolation of known properties of isozymes onto their orthologs [14,15,16]. In contrast to the laccases from fungi of the Polyporales order, less is known about laccases from fungi of the Agaricales order, and almost nothing is known about laccases from other Basidiomycetes.

Here, we present a study of laccases from *Peniophora lycii* (Pers.) Hoehn. & Litsch. belonging to the Peniophoraceae family of the Russulales order. The Russulales are one of the recently elucidated lineages in the homobasidiomycetes [17]. This order is very morphologically diverse and contains saprotrophic, ectomycorrhizal, root-parasitic, and insect-symbiotic fungi. Within this order, Peniophoraceae form a well-supported clade containing primarily saprotrophic fungi. *P. lycii* is a resupinate lichen-like species that usually colonizes dead branches of deciduous and coniferous trees all over the world.

Currently, only 11 published articles can be found about laccases from fungi of the *Peniophora* genus [18,19,20,21,22,23,24,25,26,27,28]. Worse than that, almost all these works are strictly technically oriented and use only crude laccase preparations: only in three works [20,22,28] laccases were purified; only in four works [20,21,25,26] used fungi were identified to the species level; only in two works [21,29] fungi were properly genetically barcoded; and only in three works [25,28,29] either full or partial sequences of used laccases were obtained. This situation enormously complicates any comparative studies and raises serious reproducibility issues. Additionally, although currently three genomes of *Peniophora* spp. are available, no phylogenetic study of their laccases has been performed.

In this article, two laccases isozymes of *P. lycii* LE-BIN 2142 were isolated and characterized. Further, using available genome sequences of *Peniophora* spp., phylogenetic relationships among their laccases were reconstructed and orthology groups were determined. These groups were further used for systematization of the available data about laccases of *Peniophora* spp. and as a guide for the determination of sequences for the isolated laccases of *P. lycii* LE-BIN 2142.

## 2. Materials and Methods

### 2.1. Fungal Strain and Culture Conditions

The fungal strain *Peniophora lycii* LE-BIN 2142 was obtained from the Komarov Botanical Institute Basidiomycetes Culture Collection (LE-BIN; St. Petersburg, Russia). The sequence of its ITS1-5.8S rRNA-ITS2 region is available at the NCBI GenBank accession JX046435. In the laboratory, the fungal mycelium was stored on wort-agar slants at 4 °C.

To obtain the starting inoculum, the fungus was cultivated in 750 mL Erlenmeyer flasks with 200 mL of glucose–peptone (GP) medium (per 1 L of dH_2_O): 3.0 g peptone, 10.0 g glucose, 0.6 g KH_2_PO_4_, 0.4 g K_2_HPO_4_ × 3H_2_O, 0.5 g MgSO_4_ × 7H_2_O, 0.5 g CaCl_2_, 50 mg MnSO_4_ × 5H_2_O, 1 mg ZnSO_4_, and 0.5 mg FeSO_4_. The cultivation was carried out statically for 15–18 days in the dark at 26–28 °C. The inoculum was obtained by disruption of the mycelium with ceramic beads; all inoculations were performed with 25 mL of disrupted mycelium.

For optimization of laccase production, four different media were used. Medium A had the same composition as the GP medium with an addition of 0.15 g·L^−1^ of CuSO_4_ × 5H_2_O. Medium C was different from medium A only in the concentrations of peptone (1 g·L^−1^) and glucose (20 g·L^−1^). Medium B and medium D had the same compositions as medium A and medium C, respectively, but peptone was substituted with tryptone.

All cultivations were performed in 750 mL Erlenmeyer flasks containing 200 mL of the corresponding medium. The fungus was cultivated by the submerged method on a circular shaker (180 RPM) at 27 °C for 21 days in the dark.

Laccase activity was measured spectrophotometrically using a Lambda 35 spectrophotometer (PerkinElmer, Waltham, MA, USA) with 10 mM catechol (Sigma, St. Louis, MO, USA) as a substrate (ε_410_ = 740 cm^−1^·M^−1^) in 0.1 M Na-acetate buffer pH 4.5.

### 2.2. Purification of Laccases

For laccase purification, the protocol described in [30] was modified. All procedures were performed at 4 °C. Culture filtrate (6400 mL) was 90% saturated with (NH_4_)_2_SO_4_, and the precipitate was separated by filtration (Whatman No. 1 filter paper), re-suspended in dH_2_O, and dialyzed against dH_2_O overnight. At the next stage, the preparation was stirred with DEAE-cellulose for 20 min, and the proteins were desorbed twice with 200 mM potassium phosphate buffer (KPB) pH 6.5. The resulting preparation was dialyzed against 5 mM KPB pH 6.5 and loaded on a column packed with 25 mL of DEAE-Toyopearl 650M (Tosoh, Tokyo, Japan) and equilibrated with 5 mM KPB pH 6.5. Proteins were eluted by 150 mL of 50 mM KPB pH 6.5. For further purification, fractions with laccase activity were dialyzed against 20 mM KPB pH 6.5 and subjected to FPLC size-exclusion chromatography on a Superdex 75 (26/60) column (GE Healthcare Life Sciences, Chicago, IL, USA) equilibrated with 20 mM KPB pH 6.5. Fractions with different laccase izoenzymes were transferred to 5 mM citrate-phosphate buffer pH 5.0 by dialysis and purified by an additional stage of ion-exchange chromatography on a DEAE-Toyopearl 650M column equilibrated with 5 mM citrate-phosphate buffer pH 5.0. Proteins were eluted by a linear gradient of 5–20 mM of the same buffer.

### 2.3. Gel Electrophoreses, Isoelectric Focusing, and MALDI-TOF/TOF MS Identification

The SDS–PAGE was performed according to Laemmli [31] in a Mini-PROTEAN 3 device (Bio-Rad, Hercules, CA, USA). Protein bands were stained with Coomassie Brilliant Blue R-250 (Sigma, St. Louis, MO, USA). The PageRuler Prestained Protein Ladder (Thermo Fisher Scientific, Waltham, MA, USA) with a range of 10–200 kDa was used as a standard.

The analytical isoelectric focusing on polyacrylamide gel (IEF-PAGE) was carried out using Servalyte 3–5 ampholytes (Serva, Heidelberg, Germany) on a Mini IEF Cell (Bio-Rad, Hercules, CA, USA). Protein bands were stained with Coomassie Brilliant Blue R-250 (Sigma, St. Louis, MO, USA). The protein mixture from the Amersham IEF Calibration Kit Low-Range pI (pH 2.5–6.5) (GE Healthcare UK, Amersham, UK) was used as a standard.

Two-dimensional gel electrophoresis (2D-GE) was performed on a Protean II xi 2-D Cell system (Bio-Rad, Hercules, CA, USA). The isoelectric focusing was carried out using Servalyte 3–10 ampholytes (Serva, Heidelberg, Germany), and the subsequent electrophoresis was performed in a gradient SDS polyacrylamide gel (7.5–25%). The visualization of proteins was performed with AgNO_3_ staining.

For the MALDI-TOF/TOF MS identification, the proteins were seized from the gel and digested with the trypsin. The obtained peptides were spotted on a MALDI target plate and analyzed by MALDI-TOF/TOF spectrometry on a Ultraflex II MALDI-TOF/TOF mass spectrometer (Bruker Daltonics, Bremen, Germany) equipped with a UV laser (Nd) in a positive ion regimen with a reflectron for peptide fingerprinting. The fragmentation spectra were obtained using a tandem regimen of the device, and the accuracy of measurement of fragmented ions was no less than 1 Da. The mass spectra were processed using the FlexAnalysis 3.3 program (Bruker Daltonics, Bremen, Germany). The search for proteins corresponding to MALDI-TOF/TOF MS data was carried out with Mascot Peptide Mass Fingerprint in the fungal subset of NCBI non-redundant protein sequences, taking into account possible oxidation of methionine and modification of cysteine residues. The search with combined data of the peptide masses and peptide fragmentation was performed with Biotools 3.2 (Bruker Daltonics, Bremen, Germany). Additionally, sequences of the peptides individually derived from the fragmentation data were analyzed by an in-house database of *Peniophora* spp. laccases (see “Phylogenetic Analysis” section) or BLAST NCBI using the fungal subset of the GenBank. For identification of the occupied glycosylation sites, laccase samples were treated with Endo H (Sigma, St. Louis, MO, USA) and subjected to MALDI-TOF/TOF MS/MS, as was previously described in [32].

### 2.4. Laccase Characterization

The UV–visible absorption spectra of the laccases (1.0 mg·mL^−1^) in 50 mM potassium phosphate buffer pH 6.5 were recorded with a Lambda 35 spectrophotometer (PerkinElmer, Waltham, MA, USA) in a 1 cm quartz cell at 25 °C in the range of 200–800 nm.

For the Azure B assay [33], 10 μL of the enzyme sample with a concentration of 0.1 mg·mL^−1^ was added to the reaction mixture consisting of 900 μL of 0.02% Azure B (AppliChem, Darmstadt, Germany) dissolved in 0.1 M sodium acetate buffer pH 4.5 and 100 μL of 10 mM hydroxybenzotriazole (HOBt, Sigma, St. Louis, MO, USA). The reaction was carried out at 25 °C for 24 h. Laccases from *Trametes hirsuta*, *Antrodiella faginea*, and *Steccherinum murashkinskyi* obtained in previous studies were used as control enzymes with known redox potential [33,34].

The effect of pH on the laccase activity was studied by measuring the enzyme activity in citrate-phosphate buffers (pH 2.6–6.5). Measurements were performed using the following substrate concentrations: 0.1 mM for sinapic and ferulic acids; 1mM for ABTS, gallic acid, and 2,6-DMP; 5 mM for guaiacol; and 10 mM for catechol. The molar extinction coefficients were 740 cm^−1^·M^−1^ at 410 nm for catechol [35], 29,500 cm^−1^·M^−1^ at 436 nm for ABTS [13], 35,645 cm^−1^·M^−1^ at 470 nm for 2,6-DMP [36], 26,600 cm^−1^·M^−1^ at 470 nm for guaiacol [37], 4610 cm^−1^·M^−1^ at 385 nm for gallic acid [38], 14,640 cm^−1^·M^−1^ at 306 nm for sinapic acid [39], and 12,940 cm^−1^·M^−1^ at 314 nm for ferulic acid [33]. All measurements were performed in triplicate.

Thermostability of laccases was measured after pre-incubation of the enzyme samples (0.1 mg·mL^−1^) in 50 mM KPB pH 6.5 at 70 °C, and residual activity was assayed with ABTS as a substrate.

For the dye decolorization capacity assay, Remazol Brilliant Blue R (RBBR), Bromocresol Green (BCG), Phenol Red (PR), Reactive Black 5 (RB5), Indigo Carmine (IC), and Congo Red (CR) were dissolved in water in a concentration of 1 mM. The reaction mixture contained 100 μL of dye solution, 1900 μL of buffer, and 10 μL of laccase solution with a concentration of 1 mg·mL^−1^. Sodium acetate buffer pH 4.5 was used for all dyes with the exception of CR. For the CR acetate buffer, pH 5.5 was used as this compound can change the color in the pH range of 3.5–5.2. Absorbance spectra were recorded before addition of the enzyme and after 2 and 24 h of incubation at 25 °C. The degree of dye decolorization was calculated as the relative difference in absorbance at characteristic wavelengths: 595 nm for RBBR and RB5, 615 nm for BCG, 430 nm for PR, 610 nm for IC, and 490 nm for CR.

### 2.5. DNA Extraction, PCR Amplification, and Sequencing

For DNA extraction, the fungal mycelium was ground in liquid nitrogen, and total DNA was extracted using the DNeasy Plant Mini Kit (Qiagen, Hilden, Germany), according to the manufacturer’s instructions.

To amplify the *lac5* gene, the following primers were used: F1 (5′-GCGTCTCTCTTTCACGTTAC-3′), and R1 (5′-TTAAAGAGAGTTGGGATTGGC-3′). These primers were designed based on the alignment of the nucleotide sequences of the laccases from the 5th orthology group (see Section 3.1). The amplification was performed with the Taq PCR kit (Evrogen, Moscow, Russia) and included: 1 cycle of 3 min at 95 °C; 30 cycles of (30 s at 95 °C, 30 s at 55 °C, and 150 s at 72 °C); and 1 cycle of 5 min at 72 °C. Obtained PCR products of ∼2300 bp were purified from the agarose gel by the QIAquick Gel Extraction Kit (Qiagen, Hilden, Germany) and sequenced by the standard Sanger sequencing method. The amplification primers were used at the first round of sequencing. The second round of sequencing was performed with the following primers: F2 (5′-CTAGGCACTACAAGACTCA-3′), and R2 (5′-TGCCGATAGATACCACATC-3′), designed based on the sequences obtained in the first round. All four obtained sequences were manually assembled. The exon–intron structure was predicted using Augustus software [40] and manually curated by alignment with known laccases [13]. The annotated consensus sequence was deposited into GenBank under the accession number MW172826.

### 2.6. Phylogenetic Analysis

To collect laccase sequences, the genomes of *Peniophora* sp. CONTA v1.0 and *Peniophora* sp. v1.0 were extracted from the JGI (Joint Genome Institute) portal [41], and the genome of *Peniophora* sp. CBMAI was extracted from the GeneBank database [42]. The search for laccase genes was performed using both existing functional annotations and a BLAST [43] search against known laccases [13]. As a result, 53 non-redundant laccase sequences were obtained. Since, in all genomes, protein identifiers and names of gene models are in one-to-one relationships, in the following, text protein identifiers, as the most concise, were chosen to refer to the obtained genes.

For the phylogenetic analysis, codon-based multiple sequence alignment of protein-coding sequences (CDS) was generated using the MUSCLE algorithm (File S1) [44,45]. The ambiguous alignment regions were removed with the guidance of the Gblocks (v 0.91b) program (File S2) [46]. The best fitting model of the sequence evolution was determined using jModelTest2 software [47] with 11 substitution schemes. The model selection was computed using the Akaike information criterion (AIC), and the GTR+GAMMA model was selected. The phylogenetic tree was constructed under the maximum likelihood criterion (ML) with the RAxML-HPC BlackBox (v 8.2.10) program [48] at the CIPRES Science Gateway [49]. The reliability for the internal nodes was assessed using the bootstrapping method (1000 bootstrap replicates).

### 2.7. Bioinformatics and Visualization

SignalP 5.0 Server was used to predict the signal peptide [50]. The glycosylation sites were predicted with NetNGlyc 1.0 (http://www.cbs.dtu.dk/services/NetNGlyc/) [51]. The SWISS-MODEL server was used for homology modeling of the laccase structures [52]. CCP4MG [53] was used to construct ribbon diagrams of T1 centers of laccases.

## 3. Results and Discussion

### 3.1. Orthology Groups of Laccases from Fungi of Peniophora Genus

**Figure 1:**
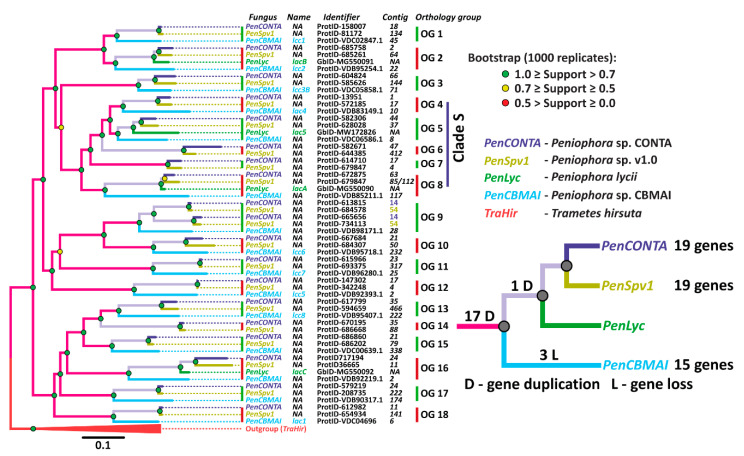
The phylogenetic relationships among laccases of *Peniophora* spp. The clades which form orthology groups are designated as OG. The clade containing laccases secreted by *P. lycii* (Lac5, Lac7, LacA) is designated as Clade S. For the laccases obtained from sequenced genomes, protein IDs (ProtID) are used as gene identifiers, while for other laccases, GenBank accession number (GbID) are provided.

Since no published information is available about the laccase multigene family in fungi of the *Peniophora* genus, at the first stage of our investigation, a search of laccase genes in the publicly available genomes of these fungi was performed. As a result of this search, it was demonstrated that fungi of the *Peniophora* genus possess from 15 to 19 non-allelic laccase genes (Figure 1). Almost all found laccase genes were located on different contigs. The exception was ProtID 613815 and ProtID 665656 from *Peniophora* sp. CONTA and ProtID 684578 and ProtID 734113 from *Peniophora* sp. v1.0. This suggests that in the genomes of *Peniophora* spp., almost all laccase genes either locate on different chromosomes of the fungi or on the same chromosome at a significant distance from each other.

On the phylogenetic tree (Figure 1), the laccase genes of *Peniophora* spp. form 18 clades containing one gene from each fungus. The exceptions are clades 6, 7, and 14 lacking the gene from *Peniophora* sp. CBMAI, and clade 9 containing two genes from *Peniophora* sp. CONTA and *Peniophora* sp. v1.0. For further discussion, both sequences of *P. lycii* laccases previously obtained by us—*lacA*, *lacB*, and *lacC* [13,54]—and the sequence obtained in the current study—*lac5*—were placed on the tree.

The topology of the phylogenetic tree indicates that the process of duplication of the ancestral laccase gene began before the splitting of the evolutionary branch leading to fungi of the *Peniophora* genus into different species. Moreover, after the division into species, both gene duplications and losses were rare events. The loss of laccase genes is observed only in the evolutionary line leading to *Peniophora* sp. CBMAI (clades 6, 7, and 14), and duplication occurs only in the closest common ancestor for *Peniophora* sp. CONTA and *Peniophora* sp. v1.0 (clade 9). It should be noted that a similar picture of the evolution of laccase genes—early multiple duplication with subsequent retention of an almost unchanged number of genes—was also described for fungi from the Core and Residual Polyporoid clades of the Polyporales order [13,55,56,57]. Similar to the Polyporales order, since almost all detected gene duplications occurred in the common ancestor of all *Peniophora* spp., all 18 clades on the constructed tree can be regarded as well-defined orthology groups.

Currently, only for *Peniophora* sp. CBMAI data about the genome, transcriptome, secretome, and the main secreted laccase are available [19,22,29,58]. Based on the transcriptomic data, it was previously shown that *Peniophora* sp. CBMAI expressed at least nine laccase genes—*lcc1-8* and *lac1* [29,58]. However, the exoproteomic study demonstrated secretion of the only one laccase isozyme—Lac1 [29], which belongs to the 18th orthology group. This isozyme was purified and characterized in [22]. A reciprocal BLAST search with previously reported partial sequences of laccases from *Peniophora* sp. VTT D-00815 [28] and *Peniophora incarnata* KUC8836 (*pilc1*) [25] allowed us to unambiguously assign them to the 5th and 15th orthology groups, respectively.

### 3.2. Culture Conditions, Purification, Identification, and Characterization of Laccases

As the level of laccase activity in fungi is largely determined by the cultivation conditions, four variants of culture media were tested for laccase production. In all the media, CuSO_4_ was used as a laccase inductor. The media were different in the type and concentration of the nitrogen source and the concentration of glucose (primary carbon source): medium A contained 0.3% of peptone and 1% of glucose; medium B—0.3% of tryptone and 1% of glucose; medium C—0.1% of peptone and 2% of glucose; and medium D—0.1% of tryptone and 2% of glucose.

In choosing these media, the following rationales were used: (1) CuSO_4_ is a well-known, most widely used, and almost universal inductor of laccase production in fungi [5]. (2) Although the peptone-containing media are widely used for laccase production, according to the previously published data for *Peniophora* sp. NFCCI-2131, replacement of peptone with tryptone increased the laccase activity in the culture liquid by 1.7 times [27]. Additionally, for *Peniophora* sp. JS17, tryptone was also indicated as the optimal nitrogen source [24]. (3) Previously, it was demonstrated that the ratio of carbon to nitrogen sources in the medium may have a substantial effect on the laccase production [5].

For all four media, the activity increased monotonically throughout the entire cultivation period. By the 21st day of cultivation, it comprised 4 U·mL^−1^, 0.8 U·mL^−1^, 1.2 U·mL^−1^, and 0.5 U·mL^−1^ on medium A, B, C, and D, respectively. Hence, to obtain a sufficient quantity of starting material for laccase purification, the fungus was further cultivated on medium A.

**Figure 2:**
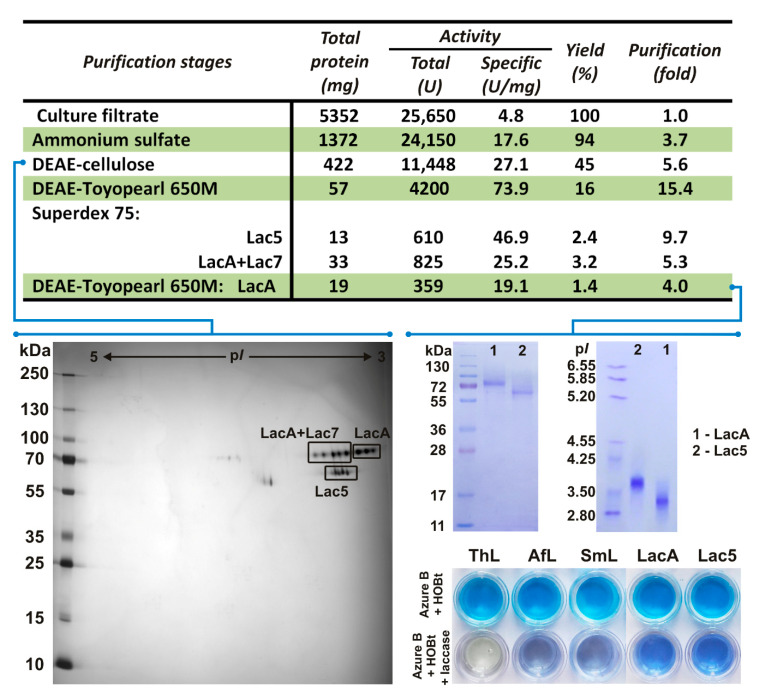
Purification scheme of *P. lycii* laccases and redox potential evaluation for purified Lac5 and LacA using the Azure B decolorization test. ThL—*Trametes hirsuta* laccase A (*E*^0^_T1_ = 780 mV), AfL—*Antrodiella faginea* laccase A (*E*^0^_T1_ = 620 mV), SmL—*Steccherinum murashkinskyi* laccase 2 (*E*^0^_T1_ = 650 mV).

At the first stage of purification (Figure 2), proteins were precipitated with (NH_4_)_2_SO_4_. Since the obtained precipitate contained a lot of pigments, the second purification stage involved batch absorption onto DEAE-cellulose. The obtained sample was desalted and concentrated. To assess the possible diversity of laccase isozymes in the sample, 2D-GE followed by MALDI-TOF/TOF MS identification was performed (Figure S1). As a result, it was shown that the sample contained three laccase isozymes (Figure 2) belonging to the fifth, seventh, and eighth orthology groups (Figure 1, Figure S1). Consequently, they were named Lac5, Lac7, and LacA. It is worth noting that for fungi of the genus *Peniophora*, the secretion of several laccases, differing in molecular weight and isoelectric point, was previously shown [20,28]. However, these studies did not establish whether the detected laccases were different isozymes (i.e., products of different genes) or isoforms (i.e., products of the same gene with different posttranslational modifications).

On the phylogenetic tree of the laccases from *Peniophora* spp. (Figure 1), it can be seen that the orthology groups secreted by *P. lycii* laccase isozymes are clustered together, forming Clade S. At the same time, among all previously reported laccases of the *Peniophora* genus, for which orthology groups could be determined, only laccase from *Peniophora* sp. VTT D-00815 (fifth orthology group) [28] belonged to this clade. The secretion of a laccase isozyme from different orthology groups by *Peniophora incarnata* KUC8836 (15th orthology group) [25] can be explained by the presence of Tween 80 and pyrene in the culture medium. Further, the secretion of laccase from orthology groups other than those contained in Clade S by *Peniophora* sp. CBMAI (18th orthology group) [29] can be a result of the very different lifestyle of this fungus, which was isolated from a coral reef in Brazil [29].

The third purification stage (Figure 2) comprised anion exchange chromatography, while the fourth stage comprised size-exclusion chromatography. Since some of the obtained fractions clearly contained two different isozymes, at the fifth stage, they were further fractionated by the second anion exchange chromatography with a different buffer system. Unfortunately, the obtainment of the pure laccase isozymes at the fifth purification stage came at a cost of diminished total specific activity and yield. As a result, two pure laccase isozymes—Lac5 and LacA—were obtained. The final amount of Lac5 (13 mg) was 1.5 times lower than that of LacA (19 mg). The yields of Lac5 and LacA were 2.4 and 1.4%, and their specific activity measured with catechol comprised 46.9 and 19.1 U·mg^−1^, respectively. The UV spectra of the obtained laccases were typical for “blue laccases” with a band at 610 nm for the T1 copper ion and a shoulder at 340 nm for the pair of T3 copper ions (Figure S2). Unfortunately, due to its insufficient quantity in the starting material, isozyme Lac7 was lost in the process of purification.

For the purified isozymes Lac5 and LacA, the determined molecular weights were 62 and 74 kDa, respectively. Both isozymes were presented by several isoforms with different p*I* values. For Lac5, the range of p*I* values was 3.7–3.8, while for LacA, this was 3.1–3.3 (Figure 2). As it was previously demonstrated, such values of molecular weight and p*I* are typical for laccases of fungi of the *Peniophora* genus [20,24,28,29] as well as for laccases of basidiomycetes in general [11]. Both laccases were relatively thermostable: the periods of half-life at 70 °C for Lac5 and LacA were 10 and 8 min, respectively.

The redox potentials of the obtained laccases were evaluated by the Azure B decolorization test, for which laccases with known redox potentials were used as controls. As it can be seen in Figure 2, LacA of *T. hirsuta* (780 mV) completely oxidized Azure B within 24 h in the presence of the HOBt mediator; LacA of *A. faginea* (620 mV) and Lac2 of *S. murashkinskyi* (650 mV) demonstrated only partial oxidation of the substrate; and both Lac5 and LacA of *P. lycii* hardly oxidized Azure B at all. Hence, it can be assumed that the values of the redox potential for both Lac5 and LacA of *P. lycii* are less than 600 mV.

### 3.3. Catalytic Activities and Dye Decolorization Capacities of the Obtained Laccases

Although the broad substrate specificity of laccases is their main advantage, it presents some problems for the unambiguous characterization of these enzymes. Since different studies use different substrates to assess laccase activity, usually, the obtained data are hard to compare with each other. To overcome this problem, in the current study, the panel of the seven most common laccase substrates was used: catechol, 2,2-azino-bis(3-ethylbenzothiazoline-6-sulfonic acid) (ABTS), 2,6-dimethoxyphenol (2,6-DMP), guaiacol, gallic acid, sinapic acid, and ferulic acid. The activities were measured at both optimal pH for the substrate and at pH 4.5.

The results of activity measurements are presented in Table 1. For all substrates but catechol and ferulic acid, the optimal pH values for Lac5 were approximately 0.5 units higher than those of LacA. For catechol and ferulic acid, optimal pH values for Lac5 and LacA were the same. Both isozymes demonstrated the highest activity toward ABTS. While Lac5 demonstrated the lowest activity toward 2,6-DMP, the lowest activity of LacA was detected for gallic acid. The main difference in the activities of Lac5 and LacA was observed for the oxidation of catechol and gallic acid, for which the activity of Lac5 was more than twice higher. Thus, it can be assumed that phenolic compounds with several hydroxyl groups are better substrates for Lac5.

It should be noted that the obtained Lac5 isozyme had similar physicochemical and catalytic properties with the previously characterized laccase from *Peniophora* sp. VTT D-00815 [28], belonging to the same fifth orthology group. This laccase was also presented by several isoforms with a p*I* range of 3.7–4.2 and a molecular weight of 63 kDa. The period of half-life at 70 °C was 15 min, and the specific activity towards guaiacol and DMP was 12% of that towards ABTS. At the same time, both Lac5 and LacA were markedly different from the previously characterized Lac1 from *Peniophora* sp. CBMAI [22], belonging to the 18th orthology group. This laccase had a lower molecular weight of 55 kDa, approximately 10 times higher specific activity towards ABTS (986.0 U·mg^−1^), and lower thermal stability (20 min half-life at 60 °C).

Currently, one of the most straightforward technical applications of laccases is decolorization of different textile dyes [59]. Being released in enormous quantities by the textile dyeing and finishing industry, textile dyes substantially contribute to the pollution of surface and groundwater resources [18]. Moreover, many synthetic dyes can act as toxic, mutagenic, allergenic, and carcinogenic agents [60]. Although synthetic dyes are very chemically heterogenic, many of them contain functional groups suitable for laccase oxidation [61].

The dye-removing capacity of the obtained laccase isozymes was assessed using a panel of six dyes: the anthraquinone dye Remazol Brilliant Blue R (RBBR), the triphenylmethane dye Bromocresol Green (BCG), the sulfonic acid dye Phenol Red (PR), the azo dye Reactive Black 5 (RB5), the indigoid dye Indigo Carmine (IC), and the diazo dye Congo Red (CR).

**Figure 3:**
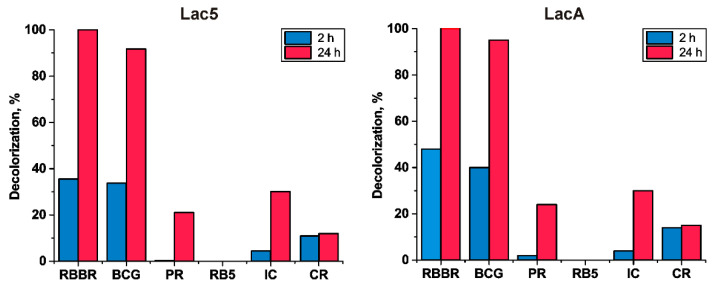
Decolorization of different dyes by *P. lycii* laccases. RBBR—Remazol Brilliant Blue R, BCG—Bromocresol Green, PR—Phenol Red, RB5—Reactive Black 5, IC—Indigo Carmine, CR—Congo Red.

As it is shown in Figure 3, both Lac5 and LacA demonstrated near identical dye-removing capacity. Within 24 hours’ time, these isozymes almost entirely decolorized such dyes as BCG and RBBR. The dyes IC, PR, and CR were decolorized only by approximately 35, 15, and 10%. In the case of RB5, there was no decolorization detected.

### 3.4. Identification of Primary Amino Acid Sequences and Structural Features of the Obtained Laccases

As it was mentioned, the primary sequence of LacA was already determined in our previous works [13,54]. To determine the primary sequence of Lac5, the specific primers were designed based on the alignment of the laccase sequences from the fifth orthology group (Figure 1). As a result, the *lac5* gene was successfully amplified and sequenced, its exon–intron structure was determined, and the coding region was conceptually translated. The obtained sequence was deposited into Gene Bank under the accession number MW172826.

**Figure 4:**
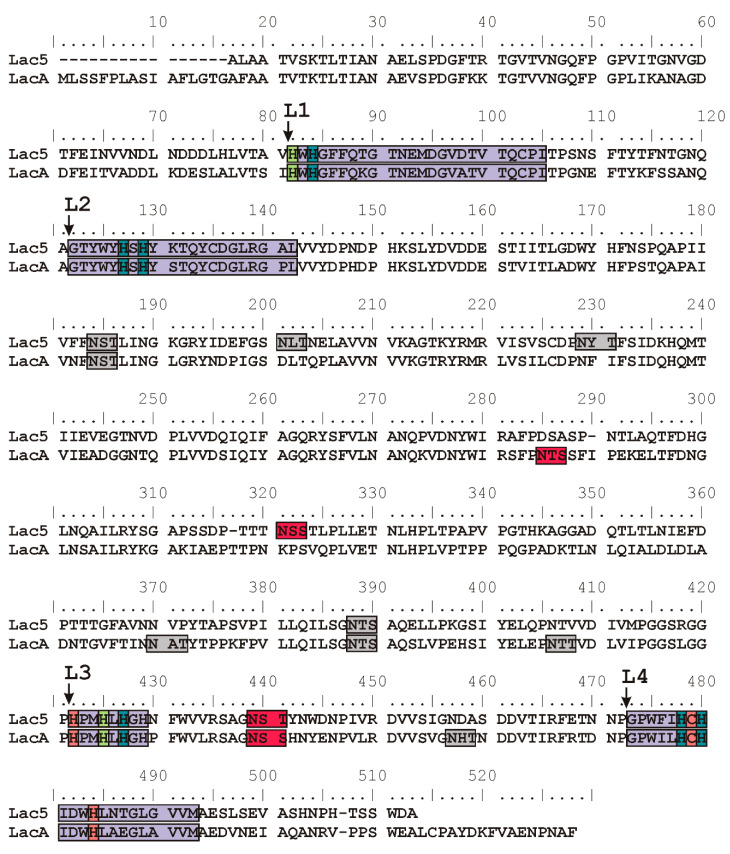
Alignment of the amino acid sequences of Lac5 and LacA from *P. lycii*. Laccase signature regions L1–L4 (according to [69]) are indicated with violet. Copper coordinating amino acid residues are indicated with coral for the T1 copper ion, leaf green for the T2 copper ion, and marine blue for the pair of T3 copper ions. Potential glycosylation sites are colored with gray and occupied glycosylation sites are colored with red.

The amino acid sequences of both Lac5 and LacA contained specific laccase signature regions L1–L4 (Kumar 2003) (Figure 4). These regions contain copper coordinating residues of the laccase active site. In both laccases, one cysteine and ten histidine residues were found which confirm that the obtained enzymes can act as the classical laccase with four copper ions in its active site. Further analysis of the Lac5 and LacA amino acid sequences revealed the presence of six and seven potential N-glycosylation sites (N-X-T/S motif), respectively (Figure 4). To reveal the actually glycosylated asparagine residues, samples of native laccases and laccases treated with Endo H were subjected to MALDI TOF/TOF mass spectrometry analysis. Deglycosylation with Endo H resulted in a decrease in molecular mass of 8% for both Lac5 and LacA. Only two sites for each laccase were confirmed to be glycosylated (Figure 4). Lac5 and LacA enzymes have the same N-glycosylated site at Asn421 (according to the LacA sequence without a signal peptide). Other occupied sites were Asn300 for Lac5 and Asn267 for LacA. Among the previously characterized laccases, modified Asn421 was found only in laccases from fungi of the Steccherinaceae family—*A. faginea* (PDB 5EHF), *S. murashkinskyi* (PDB 5E9N) and *S. ochraceum* (PDB 3T6W [62])—while modified Asn267 and Asn300 have never been observed in other basidiomycete laccases.

**Figure 5:**
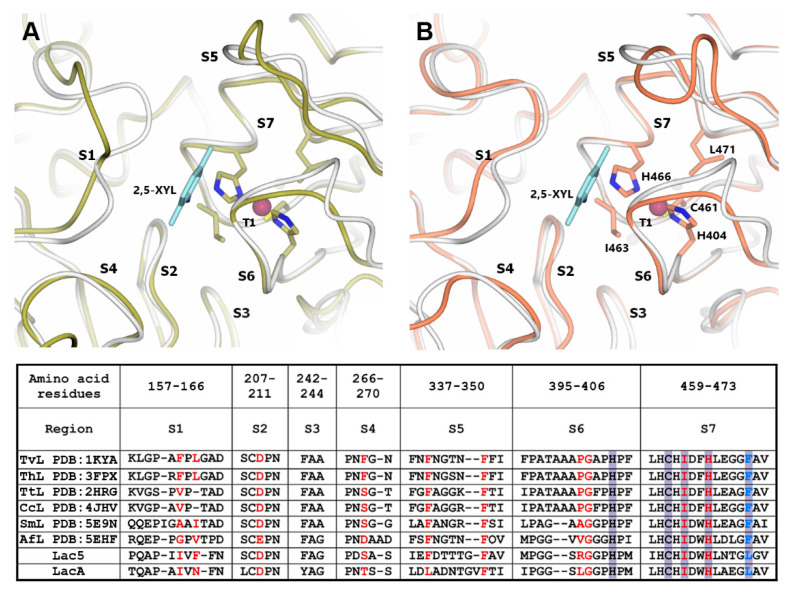
Three-dimensional models of T1 active sites and their surroundings for Lac5 (A, yellow) and LacA (B, coral). Model of *T. versicolor* laccase complexed with 2,5-xylidine (PDB 1KYA, [70]) is shown in white. Copper ions are indicated as purple spheres. Side chains of the amino acid residues from the primary coordination sphere of the T1 copper ion are shown as stick models. Molecule of 2,5-xylidine is shown with light blue color. Alignment of the amino acid sequences of regions near the T1 active sites for laccases from *T. versicolor* (TvL), *T. hirsuta* (ThL), *T. trogii* (TtL), *Coriolopsis caperata* (CcL), *S. murashkinskyi* (SmL), and *A. faginea* (AfL) and *P. lycii* laccases Lac5 and LacA is presented in the table. Amino acid residues forming the substrate binding pocket identified according to the structures of *T. versicolor* laccase complexed with 2,5-xylidine and *T. trogii* laccase complexed with p-toluate [71] are shown with red. Violet color indicates the residues from the primary coordination sphere of the T1 copper ion. Blue color indicates non-conservative residue from the primary coordination sphere of the T1 copper ion. Amino acid numbering corresponds to the LacA sequence without the signal peptide.

The oxidation of the substrate takes place at the T1 copper center of laccase [63]. Laccase catalytic activity significantly depends on the T1 copper center redox potential and also on the structure of the loops near the T1 copper center which form the substrate binding pocket [34,64,65]. To reveal the structural features of the potential substrate binding pocket of *P. lycii* laccases, homology-based modeling was performed. For both Lac5 and LacA, the high-resolution structure of *S. murashkinskyi* laccase (PDB 5E9N) was used as a template. GMQE scores were 0.83 for the Lac5 model and 0.82 for the LacA model which indicate a generally high quality of the predicted structures. It should be noted that the substrate binding pocket of laccase is formed by conservative regions (S2, S3, and S7 in Figure 5) and also by variable flexible loops (S1, S4–S6 in Figure 5) near the T1 copper center. Therefore, the obtained models could be used only for the mapping of amino acid residues potentially forming the substrate binding pocket of *P. lycii* laccases on the 3D structure of the enzyme, but the actual positions of the variable loops may differ.

Previously, it was shown that the type of amino acid residue located near the T1 copper ion (corresponding to Leu471 in LacA *P. lycii* laccase, blue color in Figure 5) may affect the value of its redox potential [66,67]. High-redox potential basidiomycete laccases (*E*^0^_T1_ > 700 mV) almost always have Phe residue in this position, while in middle-redox potential laccases (550 mV < *E*^0^_T1_ < 700 mV), Leu residue is more common [68]. Further, the value of the laccase redox potential is correlated with the structure of the variable loops near the T1 copper center [34]. Both *P. lycii* laccases have a shorter S6 loop as in all middle-redox potential laccases with known structures [34]. These findings are in agreement with the results of the Azure B test and the hypothesis that Lac5 and LacA from *P. lycii* have a middle redox potential below 600 mV.

Another interesting feature of the LacA structure is the presence of an occupied glycosylation site (Asn267) in the S4 loop. The branched carbohydrate chain attached to Asn267 may significantly affect the binding mode of the substrate as the next residue in the polypeptide chain participates in the interaction with the substrate [70]. The presence of the occupied glycosylation site at Asn267 could be the possible reason for the lower activity of LacA toward almost all substrates compared to Lac5.

## 4. Conclusions

In this work, two novel laccases of *P. lycii*—Lac5 and LacA—were purified and characterized. The molecular weights of purified isozymes Lac5 and LacA were 62 and 74 kDa and the p*I* values were 3.7–3.8 and 3.1–3.3, respectively. Both laccases demonstrated good thermostability with periods of half-life at 70 °C of 10 and 8 min for Lac5 and LacA, respectively. For all substrates but catechol and ferulic acid, the optimal pH values for Lac5 were approximately 0.5 units higher than those of LacA. Both laccases demonstrated the highest activity toward ABTS (97 U·mg^−1^ for Lac5 and 121 U·mg^−1^ for LacA at pH 4.5). Lac5 demonstrated the lowest activity toward 2,6-DMP (2.5 U·mg^−1^ at pH 4.5), while LacA demonstrated this towards gallic acid (1.4 U·mg^−1^ at pH 4.5). Since the activity of Lac5 by catechol (46.8 U·mg^−1^ at pH 4.5) and gallic acid (3.2 U·mg^−1^ at pH 4.5) was more than twice higher than that of LacA (19.1 and 1.4 U·mg^−1^ at pH 4.5, respectively), it can be assumed that phenolic compounds with several hydroxyl groups are better substrates for Lac5. Both laccases demonstrated near identical dye-removing capacity: within 24 hours’ time, they almost entirely decolorized BCG and RBBR; IC, PR, and CR were decolorized only by approximately 35, 15, and 10%; and for RB5, no decolorization was detected. The nucleotide sequences of Lac5 (GenBank MW172826) and LacA (GenBank MG550090) were determined, the corresponding amino acid sequences were deduced, and structural modeling was performed. Both laccases demonstrated structural features typical for middle-redox potential laccases. Additionally, glycosylation patterns of laccases were determined using MALDI-TOF-TOF-MS/MS. It was proposed that the presence of the Asn267-occupied glycosylation site in LacA could be the reason for the lower activity of this laccase toward almost all substrates compared to Lac5.

## Figures and Tables

**Table 1:** The optimal pH values and catalytic activities of the obtained laccases.

Substrate	Lac5			LacA		
	Optimal pH	Activity *, U·mg^−1^		Optimal pH	Activity, U·mg^−1^	
		Optimal pH	pH 4.5		Optimal pH	pH 4.5
Catechol	4.0–4.5	46.8	46.8	4.0–4.5	19.1	19.1
ABTS	3	96.9	36.8	<2.5	121.4	9.2
2,6-DMP	3.5–4.5	2.5	2.5	3.5	2.5	1.7
Guaiacol	3.5–4.0	3.8	2.9	3.0–3.5	3.6	2.3
Sinapic acid	3.5–4.0	11.2	9.1	3.0–3.5	8.0	6.2
Ferulic acid	3.5–4.0	3.1	2.6	3.5–4.0	4.1	3.1
Gallic acid	4.0	4.1	3.2	4.5	1.4	1.4

* ±SD did not exceed 5% of the given values. When not a single pH value but rather a range of pH values was optimal, the mean value of the range was used for activity measurement.

## Supplementary Materials

The following are available online at https://www.mdpi.com/2309-608X/6/4/340/s1, Figure S1: Identified peptides in *P. lycii* laccase samples. Figure S2: Laccase UV–vis spectra, File S1: Full_Alignment.aln, File S2: Trimmed_Alignment.aln.
