# Supplementary material for: Purification and Characterization of Two Novel Laccases from Peniophora lycii
Source: J Fungi (Basel). 2020 Dec 6;6(4):340. doi: 10.3390/jof6040340 (PMC7762197; doi:10.3390/jof6040340)

Figure S1. Identified peptides in *P. lycii* laccase samples.

```
                             10         20         30         40         50         60
                     ....|....| ....|....|. ...|....|. ...|....| ....|....|. ...|....|
Lac5 P_lycii         ---------- ------AL-- AATVSKTLTI ANAELS-PD- GFTRTGVTVN GQFPGPVITG
Lac5 PenSpv1         MRLSFTLPFL VSIGLEAL-- AATVSKTLTI ANTELS-PD- GFSRTGVTIN GQFPGPVITG
Lac5 PenCONTA        MRLSFTLPFL ASIGLEAL-- AATVSKTLTL ANAELS-PD- GFSRKGVTVN GQFPGPVITG
Lac5 PenCBMAI        MRISFTLPFL IS-GLEAL-- AATVSKTLTI ANAELS-PD- GFSRTGVTVN GEFPGPVITG
Lac6 PenSpv1         MRATYSLLLV SS-GLRALAV SASIIKSLVI GSTNTTYPDT DSTRTAVAIN GQFPGPVITG
Lac6 PenCONT         MRATYSLLLV SS-GLRALPV SASIIKSLVI GSTNTTYPDT DSTQTAVTIN GQFPGPVITG
Lac7 PenSpv1         MRLSFSSSLL ASLGTGAF-- AATVSQTLTI GNAVLS-PD- GYARDGVTVN GQFPGPLVSA
Lac7 PenCONTA        MRLSFLSSLL ASLGTGAF-- AATVSQTLTI GNAVLS-PD- GYARDGVTVN GQFPGPLVSA
LacA P_lycii         MLSSFPLASI AFLGTGAF-- AATVTKTLTI ANAEVS-PD- GFKKTGTVVN GQFPGPLIKA
                                                  TLTI ANAELS-PD- GFTR    VN GQFPGPLIK
                                                 QTLTI GNAVLS-PD- GYAR


                             70         80         90        100        110        120
                     ....|....| ....|....|. ...|....|. ...|....| ....|....|. ...|....|
Lac5 P_lycii         NVGDTFEINV VNDLNDDDLH LV-------T AVHWHGFFQT GTNEMDGVDT VTQCPITP--
Lac5 PenSpv1         NVGDTFAINV VNNLDDEDLH LV-------T AVHWHGFFQT GTNEMDGVDT VTQCPITP--
Lac5 PenCONTA        NVGDTFAIEV VNNLNDDDLH LV-------T AVHWHGFFQT GTNEMDGVDT VTQCPITP--
Lac5 PenCBMAI        NKGDTFEINV VNNLNDDDLH LV-------T AVHWHGFFQQ GTNEMDGVDT VTQCPITPTS
Lac6 PenSpv1         NVGDTFVINV FDDVPDDNLR LASTVVSLGT HLHWHGLFQT GTNEMDGVDR RSPSTTRTAS
Lac6 PenCONTA        NVGDTFVINV FDDVPDDNLR LASTVVSLGT HLHWHGLFQT GTNEMDGVDR RSPSTTRTAS
Lac7 PenSpv1         NVGDDFEITV VDALHDDDLA QV-------T TVHWHGFSQM GTAEMDGVDT VTQCPITP--
Lac7 PenCONTA        NVGDDFEITV VDALHDDDLA QV-------T TVHWHGFSQM GTAEMDGVDT VTQCPITP--
LacA P_lycii         NAGDDFEITV ADDLKDESLA LV-------T SIHWHGFFQK GTNEMDGVAT VTQCPITP--
                                 DDLKDESLA LV-------T SIHWH

                            130        140        150        160        170        180
                     ....|....| ....|....|. ...|....|. ...|....| ....|....|. ...|....|
Lac5 P_lycii         -SNSFTYTFN TGNQAGTYWY HSHYKTQYCD GLRGALVVYD PNDPHKSLYD VDDESTIITL
Lac5 PenSpv1         -SNSFTYTFN TGNQAGTYWY HSHYKTQYCD GLRGALVVYD PNDPHKSLYD VDDESTIITL
Lac5 PenCONTA        -SNSFTYTFN TGNQAGTYWY HSHYKTQYCD GLRGALVVYD PNDPHKSLYD VDDESTIITL
Lac5 PenCBMAI        GSNSFTYKFN SGDQAGTYWY HSHYKTQYCD GLRGALVVYD PNDPHKSLYD VDDETTIITL
Lac6 PenSpv1         -SNPFTYRFI G-NRAGTFWY YSENGTQSCD GIRGPAVVYD PNDPHRSLYD VDDETTIITL
Lac6 PenCONTA        -SNPFTYRFT G-NRAGTFWY YSENGTQSFD GIRGPAVVYD PNDPHRSLYD VDDETTIITL
Lac7 PenSpv1         -GNQFTYKFP VKDQAGTYWY HSHYKTQYCD GLRGPLVIYD PNDPQKSLYD VDDESTVWTL
Lac7 PenCONTA        -GNQFTYKFP VKDQAGTYWY HSHYKTQYCD GLRGPLVIYD PNDPQKSLYD VDDESTVWTL
LacA P_lycii         -GNEFTYKFS SANQAGTYWY HSHYSTQYCD GLRGPLVVYD PHDPHKSLYD VDDESTVITL

                            190        200        210        220        230        240
                     ....|....| ....|....| ....|....| ....|....| ....|....|. ...|....|

Lac5 P_lycii         GDWYHFNSPQ APIIVFFNST LINGKGRYID EFGSNLTNEL AVVNVKAGTK YRMRVISVSC
Lac5 PenSpv1         GDWYHFNSPQ APIIVFFNST LINGKGRYID EFGSNFANEL AVVNVKAGTK YRMRVISMSC
Lac5 PenCONTA        GDWYHFNSPQ APIIVFFNST LINGKGRYID EFGSNLTNEL AVVNVKAGTR YRMRLISMSC
Lac5 PenCBMAI        GDWYHFNSPQ APIIVYFNST VINGKGRYID EFGSNLTNEL AVVNVKAGTR YRMRLISMSC
Lac6 PenSpv1         ATWYQLNSPE SPQSGSFNPT LINGKGRDSV AFGENLANEL SVVSVEAGTR YRMRLISMSC
Lac6 PenCONTA        ATWYQLNSPE SPQSGSFNPT LINGKGRDSV AFGENLANEL SVVSVEAGTR YRMRLISMSC
Lac7 PenSpv1         SDWYHFPSPQ APIIVFFNST LINGKGRYID AFGENLTNEL AVVNVVAGTR YRIRLVSMSC
Lac7 PenCONTA        SDWYHFPSPQ APIIVFFNST LINGKGRYID AFGENLTNEL AVVNVVAGTR YRIRLVSMSC
LacA P_lycii         ADWYHFPSTQ APAIVNFNST LINGLGRYND PIGSDLTQPL AVVNVVKGTR YRMRLVSILC

                            250        260        270        280        290        300
                     ....|....| ....|....| ....|....| ....|....| ....|....|. ...|....|
Lac5 P_lycii         DPNYTFSIDK HQMTIIEVEG TNVDPLVVDQ IQIFAGQRYS FVLNANQPVD NYWIRAFPDS
Lac5 PenSpv1         DPNYTFSIDK HQMTIIEVEG TNVDPLVVDQ MQIFAGQRYS FVLDANQPVD NYWIRAFPDS
Lac5 PenCONTA        DPNYTFSIDK HQMTIIEVEG TNVDPLVVDQ IQIFAGQRYS FVLDANQPVD NYWIRAFPDS
Lac5 PenCBMAI        DPNYTFSIDK HQMTIIEVEG TNVQPLEVDQ IQIFAGQRYS FVLNANQPVD NYWIRAFPDS
Lac6 PenSpv1         DPYSMYTLYV HQMTIIEVEG TNVEPLVVDQ IQISPGQRYS FVLNANQPVD NYWIRAFPGS
Lac6 PenCONTA        DPSHTFSIDK HQMTIIEVEG TNVEPLVVDQ IQISPGQRYS FVLNANQPVD NYWIRAFPGS
Lac7 PenSpv1         DPNFIFSIDN HQMTIIEVEG TNVQPLVVDQ IQILAAQRYS FVLNANQPVD NYWIRAFPES
Lac7 PenCONTA        DPNFIFSIDN HQMTIIEVEG TNVQPLVVDQ IQILAAQRYS FVLNANQPVN NYWIRAFPES
LacA P_lycii         DPNFIFSIDQ HQMTVIEADG GNTQPLVVDS IQIYAGQRYS FVLNANQKVD NYWIRSFPNT
```

Figure S1 (continued)

```
                         310        320        330        340        350        360
              ....|....| ....|....| ....|....| ....|....| ....|....|. ...|....|
Lac5 P_lycii  ASPN-TLAQT FDHGLNQAIL RYSGAPSSDP TTT-NSSTLP LLETNLHPLT PAPVPGTHKA
Lac5 PenSpv1  ASPN-TLAQT FDHGLNQAVL RYSGAPSSDP TTT-NSSTLP LIETNLHPLT PAPVPGTHQA
Lac5 PenCONTA ASPN-TLAQT FDHGLNQAIL RYSGAPSSDP TTT-NSSTLP LIETNLHPLT PAPVPGTHEA
Lac5 PenCBMAI ASPN-TLAQT FDHGLNQAIL RYAGAATADP TTT-NSSTLP LLETNLHPLT STPVPGTHAA
Lac6 PenSpv1  TGTN-TLPQA SEHGLSQAIL RYSGASLADP ITTTTSPTLP LLETNLHPLK PTPVPGVLAV
Lac6 PenCONTA TGTN-TLPQA SEHGLNQAIL RYSGASLADP ITTTTSPTLP LLETNLHPLK PTPVPGVLAV
Lac7 PenSpv1  ASPN-TLAQT FDHGLNQAIL RYKGALATDP TTT-NSSTLP LVETDLHPLT PLPVPGKHTA
Lac7 PenCONTA ASPN-TLAQT FDHGLNQAIL RYKGAPATDP TTT-NSSTLP LVETDLHPLT PLPVPGKHAA
LacA P_lycii  SSFIPEKELT FDNGLNSAIL RYKGAKIAEP TTPNKPSVQP LVETNLHPLV PTPPPQGPAD
                 IPEKELT FDNGLN          PSSDP TTT-NSSTLP LLETNLHPLT PAPVPGTHK
                                            DP TTT-NSSTLP LVETDLHPLT PLPV

                         370        380        390        400        410        420
              ....|....| ....|....| ....|....| ....|....| ....|....|. ...|....|
Lac5 P_lycii  GGADQTLTLN IEFDTTTG-F AVNNVPYTAP SVPILLQILS GNTSAQELLP KGSIYELQPN
Lac5 PenSpv1  GGADQTLTLN IEFDTTTG-F AVNNVQYAAP SVPILLQILS GNTSAQELMP KGSIYELQPN
Lac5 PenCONTA GGADQTLTLN IEFDTTTG-F AVNNVQYAAP SVPVLLQILS GNTSAQELMP KGSIYELQPN
Lac5 PenCBMAI GKADQTLTLN IEFDDATG-F AVNNVAYAAP SVPILLQILS GNTSAAELMP KGSIYELQPN
Lac6 PenSpv1  GGADSTLTLN LTSGEMVD-F SANNVPYTAL DVPILRRNRS DVTAAQELTS RGGAHEPRSN
Lac6 PenCONTA GGADSTLTLN LTSGEMVD-F AANNVPYTAP DVPILRRTRS DITTAQELTS RGGAHEPQSN
Lac7 PenSpv1  GGADKTLTLN IEFNDTTG-F YVNNVQYMGP SVPVLLQILS GNKTAQQLMP AGSIYGLDPN
Lac7 PenCONTA GGADKTLTLN IEFNDTTG-F YVNDVQYMGP SVPVLLQILS GNKTAQQLMP AGSIYGLDPN
LacA P_lycii  KTLNLQIALD LDLADNTGVF TINNATYTPP KFPVLLQILS GNTSAQSLVP EHSIYELEPN
                                                                            DPN

                         430        440        450        460        470        480
              ....|....| ....|....| ....|....| ....|....| ....|....|. ...|....|
Lac5 P_lycii  TVVDIVMPGG SRGGPHPMHL HGHNFWVVRS AGNSTYNWDN PIVRDVVSIG NDASDDVTIR
Lac5 PenSpv1  TVVDIVMPGG SRGGPHPMHL HGHNFWVVRS AGNSTYNWDN PIVRDVVSIG NDATDDVTIR
Lac5 PenCONTA TVVDIVMPGG SRGGPHPMHL HGHNFWVVRS AGNSTYNWDN PIVRDVVSIG NDATDDVTIR
Lac5 PenCBMAI TVVDIVMPGG SRGGPHPMHL HGHNFWVVRS AGNSTYNWDN PIVRDVVSIG NDATDDVTIR
Lac6 PenSpv1  SVIDLVVPGA SLAG-QSVHL HGHNFWVIRS AGNSSYNWDN PIVRDVVSVG NDAMDNVTIR
Lac6 PenCONTA SVIDLIVPGA SLAG-QSMHL HGHNFGVIRS AGNSTYNWDN PIVRDVVSIG NDALDNVTIR
Lac7 PenSpv1  TTVDLVIPGG SRGGPHPMHL HGHHFWVIRS ANNATYNFDN PIYRDTVNIG NSSTDEVTVR
Lac7 PenCONTA TTVDLVIPGG SRGGPHPMHL HGHHFWVIRS ASNSTYNFDN PIYRDTVNIG NSSSDLVTVR
LacA P_lycii  TTVDLVIPGG SLGGPHPMHL HGHPFWVLRS AGNSSHNYEN PVLRDVVSVG NHTNDDVTIR
              TTVDLVIPGG SRGGPHPMHL HGHNFWVVRS TYNWDNPIVR
                           GGPHPMHL HGHHFWVIR  TYNFDNPIYR
                                                 NYENPVLR

                         490        500        510        520        530        540
              ....|....| ....|....| ....|....| ....|....| ....|....| ....|....|
Lac5 P_lycii  FETNNPGPWF IHCHIDWHLN TGLGVVMAES LSEVASHNPH -TSSWDA--- -------------
Lac5 PenSpv1  FETNNPGPWF IHSHIDWHLN TGLGVVMAES LSEVASHNPH -TSSWDALCP AYNNFVSANP NSL
Lac5 PenCONTA FETNNPGPWF IHCHIDWHLN TGLGVVMAES LSEVASHNPH -TSSWDALCP AYNNFVSANP NSL
Lac5 PenCBMAI FETNNPGPWF IHCHIDWHLN TGLGVVMAES LANVASANPH -TSSWDALCP AYNNFVAADP NSL
Lac6 PenSpv1  FETNR-LLLD TGCQVDLNVD TGSDVVMAKS PAKLASDVNV ---KKCALIL NVHLVVHHTS GP-
Lac6 PenCONTA FETKD-LLLN TVSLIGT--- ---------- ---------- ---------- ---------- ---
Lac7 PenSpv1  FVTDNPGPWF FHCHIDWHLN TGLGVVMAES VSEVAAANPN IPSSWDALCP AYNDYVASHP NSL
Lac7 PenCONTA FVTDNPGPWF FHCHIDWHLN TGLGVVMAES VSEVAAANPN -PSSWDALCP TYNDYIASHP NSL
LacA P_lycii  FRTDNPGPWI LHCHIDWHLA EGLAVVMAED VNEIAQANRV -PPSWEALCP AYDKFVAENP NAF
```

Lac5 unique peptides   LacA unique peptides
Lac7 unique peptides   Common peptides for several laccase isoenzymes

Figure S2. Laccase UV-vis spectra

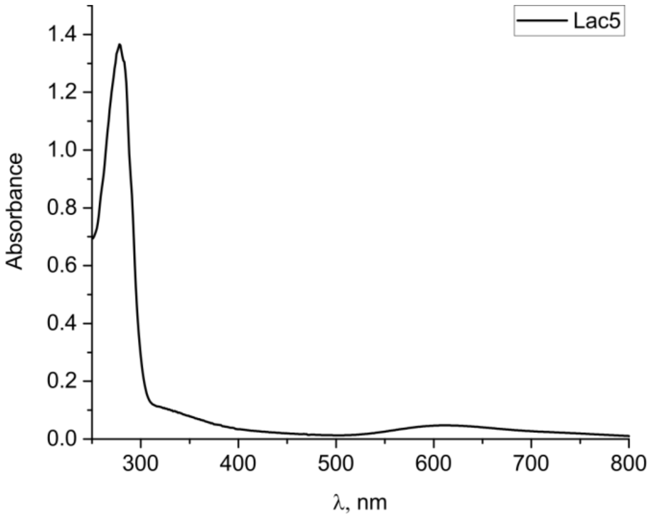

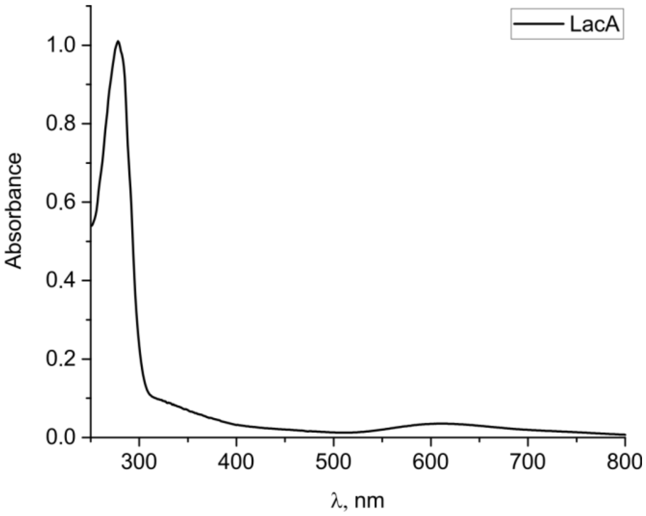

Supplement: Supplementary file 1 [file jof-06-00340-s001.zip › Supplementary_materials.pdf]
